# The Role of Macroeconomic Indicators on Healthcare Cost

**DOI:** 10.3390/healthcare8020123

**Published:** 2020-05-04

**Authors:** Lu Lin Zhou, Sabina Ampon-Wireko, Ebenezer Wireko Brobbey, Lamini Dauda, Joseph Owusu-Marfo, Arielle Doris Kachie Tetgoum

**Affiliations:** 1 Deparment of Management Science and Engineering, Jiangsu University, Zhejiang 212013, China; zll62@126.com (L.L.Z.); dabremp@gmail.com (L.D.); 5103170254@stmail.ujs.edu.cn (J.O.-M.); 5103170109@stmail.ujs.edu.cn (A.D.K.T.); 2 Ghana Education Service, Accra PMB, Accra GA 111-5469, Ghana; seekwa12@outlook.com

**Keywords:** health expenditure, tax revenue, Inflation, research and development, labor force participation

## Abstract

The progress of healthcare expenses is a striking issue for emerging nations. This is because an uncontrolled increase in healthcare expenses can push the nations to extreme poverty. The study examined the association between public health costs and macro-economic indicators within the emerging economies. Data for the study is extracted from the World Bank World Development Indicators for twenty-one (21) emerging countries spanning from 2000 to 2018. The generalized method of moments (GMM) and the Dumitrescu-Hurlin panel causality test are employed in the analysis of the study. The main findings of the study demonstrate that tax revenue and labor force participation increase public health expenses and inflation, on the other hand, showed a declining relationship. The study further reveals a u-shaped association between public health expenditure and economic growth. The interactive term between research and development and mortality rate of non-communicable diseases, reveal an increasing relationship. The study establishes that, among all the three models estimated, tax revenue, labor force participation and GDP per capita have positive effects on public health costs. Based on the findings, the study recommends governments to embark on policies that improve economic growth and tax revenue as well as stabilizing inflation. These strategic policies could boost public healthcare expenditure since it has a strong association with macroeconomic indicators.

## 1. Introduction

The health-care system plays a significant role in a nation’s development by improving population health. Notwithstanding, an increase in healthcare cost has become a concern for individuals and governments across the globe [1]. Governments are therefore making concerted effort to increase public spending for medical care with the primary goal of enhancing population health [2]. Although there is extensive literature tracking trends and factors influencing public health spending in high-income countries, studies focusing on the drivers of health spending in emerging countries remains relatively scarce [3,4,5]. Following the ground-breaking study of Newhouse [6], the determinants of healthcare expenditure have been categorized into economic and non-economic factors [7]. According to [8,9], the economic factors influencing health expenditure include per capita gross domestic product (PGDP), tax revenue, liquidity rate, inflation, trade (exports plus imports) while the non-economic variables consist of population, education and lifestyle [10]. Tajudeen [11] established that increase in macroeconomic indicators especially income is crucial for the provision of comprehensive health care that is important for promoting and maintaining population health.

However, as fastest-growing economies, emerging nations do not only attract large quantities of production but increase labor from different parts of the world. For this reason, it is crucial for the provision of quality healthcare and this will require substantial resources. Nevertheless, from available literature, there is a limited number of studies [12,13,14,15], that analyzed the effect of macroeconomic factors on public health costs. Similarly, Taskaya, Demirkiran [16] examined the association between healthcare expenditure, inflation and gross domestic capita per (GDPPc) in Turkey using data from the World Bank and Organisation for Economic Co-operation and Development (OECD) Health Database between 1975 to 2013. The study found no relationship between health expenditure, inflation rate and GDP per capita utilizing the granger causality approach.

In other study, Behera, Dash [17] employed the panel vector error correction models, fully modified ordinary least squares (FMOLS) and dynamic ordinary least squares (DOLS) to assess the effect of tax revenue, gross domestic capita GDP on health care expenditure (HCE) for the period 1980–2014. The findings show a positive and significant effect of tax revenue and per capita GDP on public health costs. Barik and Arokiasamy [18] also examined the association of health care costs between non-communicable diseases and by employing data from the health and morbidity survey it was found that non-communicable diseases upsurge health care costs. In a similar vein, Chen, Kuhn [19] concluded that the effects of non-communicable diseases are beyond mortality and ill-health which leads to huge financial consequences. Furthermore, Osondu, Aneni [20] examined the connections between health care expenses and cardiovascular disease in Florida. It was established that a reduction in total medical costs is associated with cardiovascular health. In another breath, Bajpai and Sachs [21] and Giammanco, Gitto [22] examined the effect of foreign direct investment (FDI) on health expenses in Europe. These studies, however, concluded that FDI contributes to increasing expenses of public healthcare. From the previous studies, it could be seen that even though macroeconomic indicators play an essential role in health care delivery, it has not been extensively studied and therefore cannot be ignored if sustainable development goal (SDG) 3 is to be obtained.

Given the huge size of population in emerging countries and their significance at the global level, the role of macro-economic factors presently has been a concern for most industries, including the health care sector [23]. An on-going discussion has been centered on whether macroeconomic indicators including tax revenue, inflation and GDP influence health care costs for the fulfilments of the universal health coverage. To our knowledge, few studies have used macro-economic data to examine the trend of health expenses in the emerging economies [24]. The study has taken into account the role of tax revenue, inflation and GDP on health spending in emerging nations. Previous studies concentrated on the linear relationship between economic growth and health. This study moves further to deepen the understanding by testing the nonlinear relationship between health expenses and economic growth using the Kuznets curve hypothesis. Methodologically, some studies applied estimation techniques, such as fixed effect model and unit root Behera and Dash [24], pooled mean group estimation Barkat, Sbia [25] and others Zhang, Zhang [26], Akinlo and Sulola [27], Aboubacar and Xu [28] used the generalized methods of moments procedure to investigate the factors influencing health care costs.

The study explores the role of macro-economic factors and cardiovascular diseases on public health expenditure utilizing the generalized method of moments. The motive behind the selection of the variables stems from the high recorded cases of diabetes, cardiovascular diseases and cancer in recent years Nascimento, Brant [29], particularly in emerging economies.

**Figure 1:**
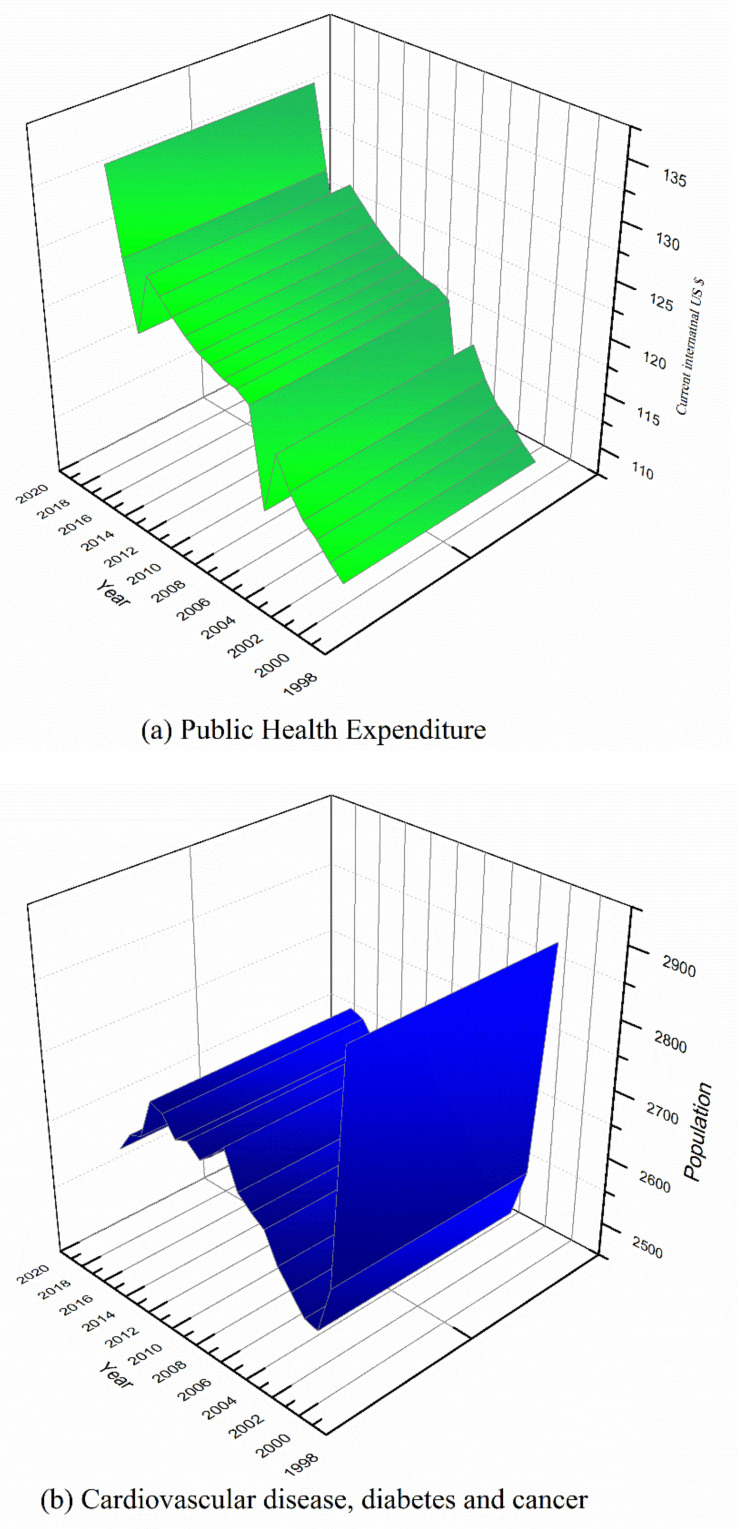
Health indicators (**a**) public health expenditure (**b**) cardiovascular disease, diabetes and cancer.

Figure 1a,b displays both the trend of public health expenditure and cardiovascular disease, diabetes and cancer from 2000 to 2018. From Figure 1a, an increasing trend of public health expenditure among the emerging economies is obvious. Thus, these countries are increasingly devoting more of their percentage (%) of GDP to support healthcare. Figure 1b reveals that cardiovascular disease, diabetes and cancer experienced a reduction from the year 2000 to 2004 and it rose steadily from 2003 up to 2016 but evidence a gradual decline until 2018. Although Figure 1b indicates a decreasing trend as of 2018, the trend can be reversible if necessary, measures are not in place. Such measures may include behavior change modifications including reducing excessive salt intake, exercising regularly and adhering to fruits and vegetable consumption and avoiding the intake of tobacco use and alcohol.

**Figure 2:**
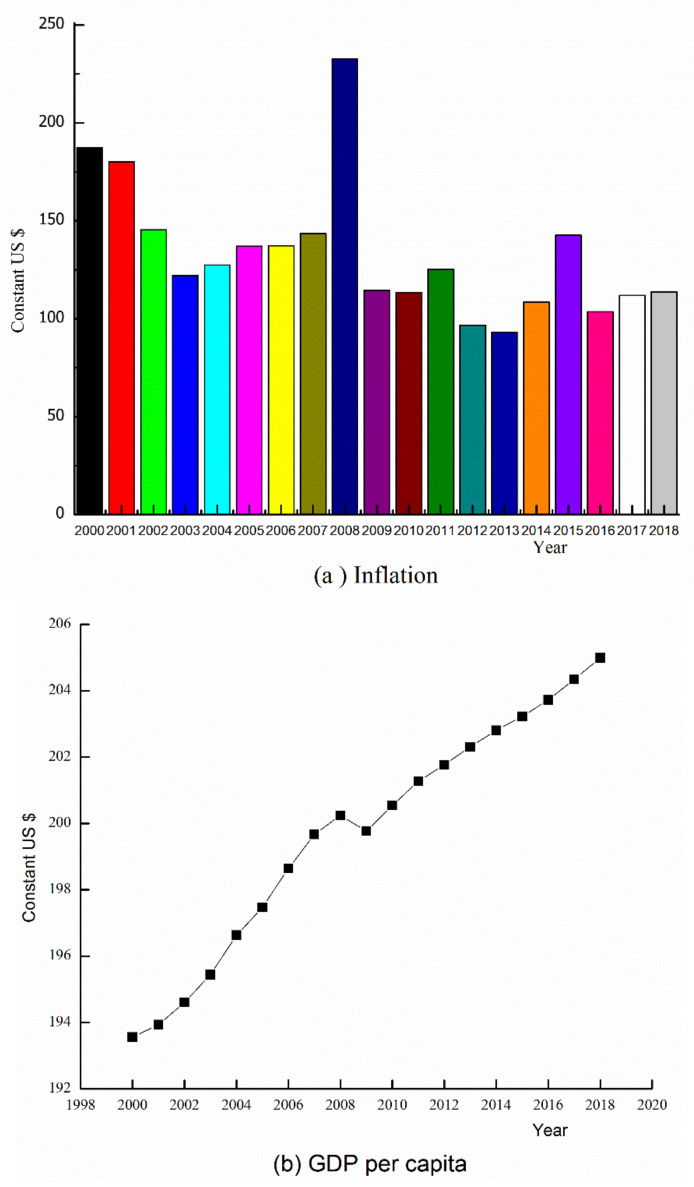

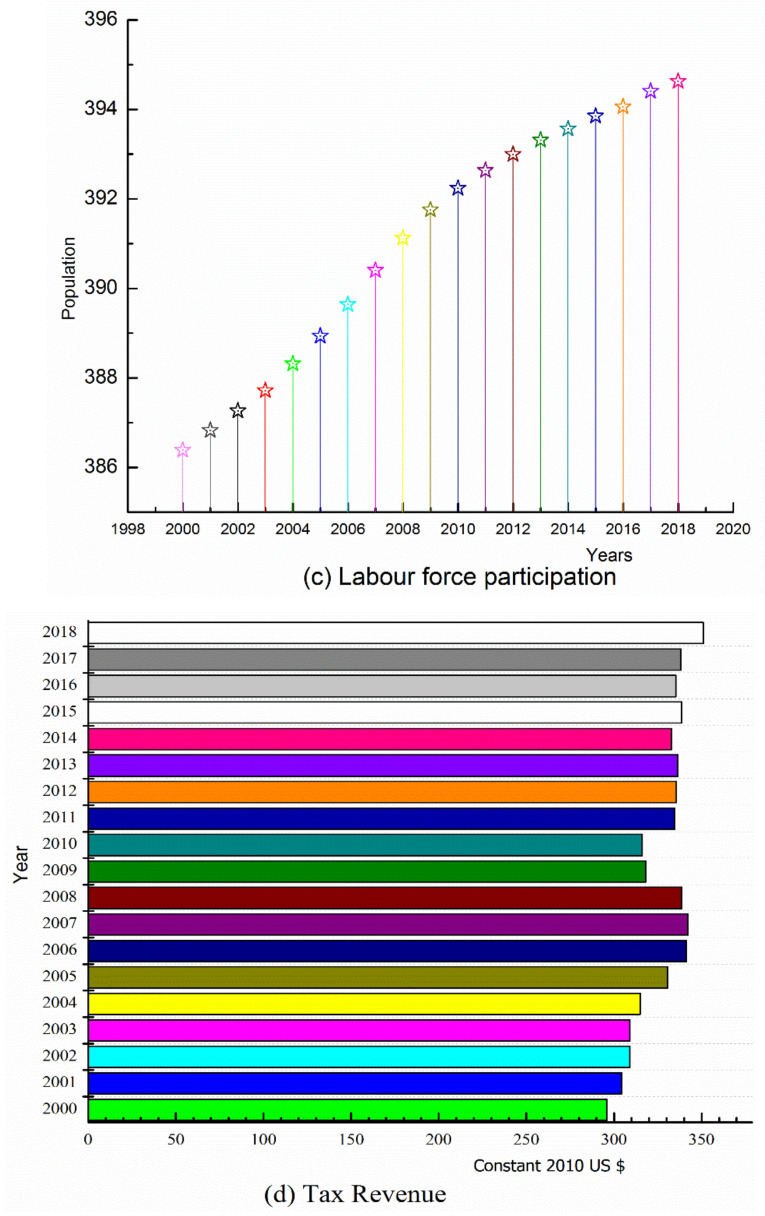
Macroeconomic indicators (**a**) inflation (**b**) GDP per capita (**c**) labor force participation (**d**) tax revenue.

In Figure 2, macroeconomic, indicators signify the behavior and productivity of price of goods and services at a particular point in time. Comparing the year 2000 to 2018, it can be seen that except for 2008, inflation experienced a sharp increase to the peak. Figure 2b evidences the trend of GDP per capita within the emerging nation nations between the years 2000 to 2018. It can be observed that the values of GDP per capita rose steadily from 2000 to 2008 but fell gradually until 2011. However, the values of GDP per capita increased sharply between 2012 and 2018. Figure 2c also depicts the trend of the percentage of the working-age population between 2000 and 2018. A critical look at Figure 2d shows the variation of compulsory levies imposed on individuals or organizations by the governments to generate income for the common good of the public. A deduction is that Tax revenue growth rate has been unstable within the period with a respective maximum and minimum value of $300 and $350.

The following sections of this study are organized as follows: Section 2 presents the methodology, data and model specification; Section 3 provides empirical results; Section 4 discusses findings and Section 5 finally concludes the study with implications for policy.

## 2. Methodology

### 2.1. Data

The dataset utilized in the study is extracted from the World Health Organization and the World Development Indicators (WDI) of the World Bank Database. The study countries include; Egypt, Indonesia, India, Pakistan, Philippines, Brazil, Columbia, Peru, Thailand, China, South Africa, Chile, Czech Republic, Greece, Russia, United Arab Emirates, Turkey, Poland, Ukraine, Bulgaria and Malaysia. emerging nations are studied because they are related to each other in terms of many socioeconomic indicators including growth rate, economic development and health challenges. The empirical analysis covers a panel data of twenty-one (21) emerging economies for 2000 to 2018 based on the availability of data. The variables for the study are presented in Table 1 below.

### 2.2. Preliminary Procedures

A preliminary test involves key essential tests prior to the selection of the best econometric technique to aid in achieving the objective of the study. The study performed the F-statistics, Hansen tests and the Arellano–Bond (AR1, AR2) test. Results from the F-statistics helped examine if the dependent variable is large and sufficient to avoid weak and instrumental bias. To test whether the model specification is right, the Hansen tests [30] is performed. Hansen test helps to choose between the many possible methods of moments estimators in a framework helps resolve the issue of heteroscedasticity and serial correlation. The study employed Arellano–Bond [31] to estimate the long-run relationship among the variables because it provides efficient results for studies with small time variance a large number of countries.

### 2.3. Model Modification

This study applied the generalized method of moments (GMM) Hansen [30] to estimate the results. GMM estimation provides a direct way to examine the specification of the model proposed. This is a vital feature about GMM procedure [30]. The GMM procedure is established on the postulation that, the error term is serially not correlated. However, following the study Ssozi and Amlani [32] the GMM model is constructed as

(1) $$\;\ln{\left(Y\right)}_{it}=\sum_{f=1}^{h}\beta_{1}\ln{\left(Y\right)}_{it-f}+\gamma_{l}\ln{\left(x\right)}_{it-l}+\delta_{i}+\varepsilon_{it}$$

(2) $$E\left[\delta_{i}\right]=E\left[\varepsilon_{it}\right]=E\;\left[\delta_{i}\varepsilon_{it}\right]=0$$

here *Y* indicates the dependent variable, *X* includes the independent and the control variables $\delta_{i}$ are the unobserved time-invariant country-specific effects whereas $\varepsilon_{it}$ is the observation error term.

To construct models for the study, we considered the following aggregate production function adapted from the work of [25]. The general form of public health expenditure is presented as:

(3) $$HCE=\int \left(GDPPC,\;INF,\;TAX,\;LAB,\;FDI,\;R\&D,Mortality\right)$$

(4) $$lnHCE_{it}=\theta_{i+}\beta_{1}lnGDP_{it}+\beta_{2}lnMortality_{it}+\beta_{3}lnFDI_{it}+\beta_{4}R\&D_{it}+\mu_{it}$$

(5) $$lnHCE_{it}=\theta_{i}+\beta_{1}lnGDP_{it}+\beta_{2}lnGDP_{it_{2}}+\beta_{3}lnMortality_{it}+\beta_{4}lnINF_{\begin{array}{c} it \end{array}}+\beta_{5}lnLAB_{it}+\beta_{6}lnTax_{it}+\mu_{it}$$

(6) $$lnHCE_{it}=\theta_{i+}\beta_{1}lnINF_{it}+\beta_{2}lnTAX_{it_{2}}+\beta_{3}lnLAB\times FDI_{it}+\beta_{4}lnR\&D\times Mortality_{it}+\beta_{5}lnFDI\times R\&D_{it}+\mu_{it}$$

where HCE is the dependent variable representing public health expenditure, with explanatory variable including the GDPPC (GDP per capita), mortality (death resulting from cardiovascular diseases, cancer and diabetes), INF (inflation), LAB (labor force participation), TAX (tax revenue), R&D (research and development), LAB×FDI (interactive term of labor and foreign direct investment), R&D×Mortality (interactive effect between research and development and death resulting from cardiovascular diseases, cancer and diabetes) and FDI×R&D (interactive term of foreign direct investment and research and development). The *β* is the vector coefficient of independent variables and *α* is the intercept which represents the country also μ denotes the error term.

The main variables of the study include public healthcare expenditure, inflation, GDP per capita, labor force participation and tax revenue. The control variables comprise Foreign Direct Investment research and developments and death resulting from cardiovascular disease, diabetes and cancer.

### 2.4. Robustness Check

To perform a robustness check, the study employed the panel ordinary least square regression. Findings from the panel ordinary least square regression will help confirm the validity of our results from the GMM estimation procedures.

## 3. Results

### 3.1. Descriptive Statistics

In Table 2, the descriptive analysis of the data set shows the mean, median, skewness, kurtosis, maximum, probability, Jarque–Bera and minimum. Kurtosis measures the flatness of the series distribution. The outcome of the analysis shows FDI and Tax revenue displayed negative skewness. Evidence from Jarque–Bera proved a normal distribution and therefore establishing the null hypothesis statement that, all the variables are normally distributed at 5% significance level. The maximum value of the analysis discloses Tax revenue as the variable with the highest values (26.512) and therefore an important variable in emerging economies while FDI is found to be the variable with the minimum value (0.000). In addition, the standard deviation exposed cardiovascular diseases as the utmost explosive variable.

### 3.2. Results from Arellano–Bond and Hansen-J Test

AR2 and Hansen-J test reject the alternative statement of the existence of autocorrelation. The results of p-values of the Hansen tests in the three models are insignificant rejecting the issue of over-placing restrictions and revealing the validity of study instruments [30]. However, it accepts the null statement of endogenous with no autocorrelation among variables hence the study concludes that the GMM procedure is valid for the study.

### 3.3. Results for Generalized Method of Moments (GMM)

Findings from Table 3 show that the association between public health costs and income is positive and significant. An indication that an upsurge in public health cost by 1.096% is associated with 1% increase in economic growth. The association between public health cost and death from diabetes, cardiovascular disease and cancer is also positive and significant. Suggesting that a rise in public health cost by 0.0943% is associated with 1% surge in diabetes, cardiovascular disease and cancer. In addition, foreign direct investment (FDI) shows a positive and significant association. This demonstrates that an escalation of public health expenditure by 0.003% is associated with a unit upsurge of FDI. Public health expenditure again has a positive and significant association with R&D.

Furthermore, the result shows a u-shaped association between public health cost and economic growth. Economic growth though insignificant showed a negative sign of 0.0262% whiles economic growth squared depicts an important and significant increase of 0.023%. The rate of death from diabetes, cardiovascular disease and cancer is positive and significant. Demonstrating that, increase in public health costs by 0.0271% could be associated with 1% surge in mortality rate from cardiovascular diseases, diabetes and cancer. Labor force participation was found to be positive and significant. Public health cost again denotes a positive significant association with tax revenue. Showing that public health expenses upsurge by 0.00211% as tax revenue grows by 1% within emerging nations.

Model (3) depicts that, the association between public health cost and inflation is positively significant. An indication that 0.108% increase in public health costs can be associated with 1% increase in inflation. Again, a positive and significant relationship exists between public health costs and tax revenue. The findings suggest that increase in public health costs by 0.108% may be associated with a unit increase of tax revenue. The interactive effect between labor force and foreign direct investment (LAB×FDI) is positively significant. The results offer evidence that an upsurge public health cost by 0.025% can be associated with LAB×FDI. In addition, the study denotes that increase in public health costs by 0.246% is associated with a 1% increase in research on diabetes, cardiovascular disease and cancer. Finally, public health expenditure has a negative association with the interaction between foreign direct investment and research. An indication that an escalation in health expenses by 0.289% is associated with a rise in FDI and R&D.

### 3.4. Robustness Check

For a robust check, the OLS regression test was performed and the result for model 1 in Table 4 reveals that, the association between public health cost and income is positive and significant. Thus, an increase in public health expenditure by 0.9387% is associated with 1% escalation of economic growth. The association between cardiovascular disease, cancer and diabetes is also positive and significant. A sign that a rise in public health cost by 0.0943% is associated with cardiovascular disease, diabetes and cancer raise. FDI shows a positive and significant coefficient demonstrating that a surge in public health costs by 0.0083% is associated with a unit upsurge in FDI. The findings depict that, public health costs is positively associated with medical research. Implying that, 0.036% surge in public health care costs is associated with R&D growth.

Model (2) shows a u-shaped association between public health expenses and economic growth. Income reveals a negative sign whiles income squared denotes a positive coefficient. The association between public health cost and death from diabetes, cardiovascular disease and cancer is significantly positive. Labor force is positive and significant. This means a rise in public health expenses by 0.42% is associated with a unit increase in labor force. Additionally, public health cost reveals a positive and significant association with tax revenue, however a negative and a significant association was seen between public health cost and inflation.

From model (3), Inflation reveals a negative and important association. Conversely, a positive and significant association is found between public health costs and tax revenue. The results indicate that increase in public health cost by 0.769% is attributed to a 1% surge in tax revenue. The association between public health cost and interaction effect between labor force and foreign direct investment (LFDI) is positively significant. An indication that, 0.0010% increase in public health cost is the result of (LAB×FDI) growth. Furthermore, public health costs is positively associated with the interactive effects of R&D×Mortality. The study denotes that increase in public health costs by 0.0558% is associated with research in diabetes, cardiovascular disease and cancer. Finally, the interaction term of foreign direct investment together with research and development (FDI×R&D) shows a positive association. Thus, 0.0099% increase in public health expenses is associated with 1% upsurge in FDI×R&D.

## 4. Discussion

The study suggests that, a u-shape relationship exists between public health cost and economic growth in emerging economies. The u-shape relationship indicates public healthcare costs possibly decrease at low-income level, reach a minimum point, and then start to increase as income increases beyond this threshold. From the results, when these countries exceed a certain level of income, they will be able to invest massively in the health sector. In another vein, the u-shape relationship exists because the resources allocated to healthcare sector at the initial stage of development may not be enough in relation to population growth. In addition, therefore, when demands for healthcare far exceeds its supply, public healthcare cost and economic growth could exhibit a u-shape relationship. To reduce the turning point countries should intensify their support for healthcare through universal health coverage, which aims to minimize barriers to access health care. This finding is in harmony with the conclusions of Spiteri, von Brockdorff [33] in European countries and Costa-Font, Hernandez Quevedo [34] in Bangladesh.

Increase in public healthcare cost in health expenses could be attributed to an up surge of death caused by diabetes, cardiovascular disease and cancer could lead to an within emerging economies because of demand for certain special equipment and training of specialists to be able to detect and treat them. To resolve this, the study explores the interactive effect of (R&D×Mortality). The findings indicate public health expenses increases as a result of the interactive effect of R&D×Mortality. This may result from the higher cost of quality medical resources needed for an in-depth study of treating diabetes, cardiovascular disease and cancer. Datta, Husain [35] and Saksena, Hsu [36] also found that death from diabetes, cardiovascular disease and cancer surge public health cost. This may be that, even though in emerging nations, research into health could be ongoing, it has not translated into decreasing the trend of some of these diseases to minimize the government’s expenditure in the healthcare system. This could be the reason why the interaction effect still surges public healthcare cost in the selected emerging economies.

In addition, the study suggests that public health expenses is influenced by labor force participation. Possible explanation could be that when there is a full labor force participation in a country, certain jobs such as mining and other industries expose their workers and surrounding communities health hazards that can lead to diseases frequent visits to the health facility. When this happens, it puts pressure on government and stakeholders for expansion in hospitals leading to healthcare expenditure and vice versa with the assumption that all other things being equal. The study corroborates with the results from [37] in the United States that concluded that the labor market and health workforce cause a rise in government healthcare spending.

Public health expenses reveal a positive and considerable relationship with Tax revenue. An indication that increases in tax revenue may increase government health expenses on healthcare. This may be related to the fact that some emerging countries that succeed in increasing public health spending could do so by raising tax revenue. In other words, revenue from tax can serve as a source of government income used to fund public services including health care [38]. Carter and Cobham [39] also confirmed that revenues generated from taxes of firms could enable governments to build more hospitals, train more health providers and deliver other required resources to enhance better health of citizens

On one hand, the results indicate healthcare costs is negatively affected by inflation in emerging countries. This means even though negative inflation on the economy, in general, may not be a good thing, its effects on healthcare could not increase healthcare costs because prices of goods and services related to health are not increasing. The negative relationship of healthcare cost relating to inflation suggests the value of healthcare costs in monetary terms as stable or decreasing because inflation is not increasing prices but rather decreasing prices. On the other hand, the chances of inflation having a negative effect on the general economic growth are obvious and this could impede income sources to the health system with an ultimately negative impact on health costs. Consistent with the conclusions of [9] in Iran and [40] in Zimbabwe that a decreasing trend between public health expenditure and inflation exist. The study therefore recommend the need for further analyses using other macro-economic variables, such as foreign aid, interest and exchange rate which are also considered to be connected to public health costs.

The results again show public health expenditure increases as medical research upsurges and this may be explained by the huge sum of money required by health sectors to study and develop vaccines and purchase drugs for previously unpreventable and untreatable terminal conditions. Public health expenses increases as Research and development medical research escalates amount of health care that people seek in their lifetime [41]. It can be inferred from the findings that public health spending accelerates as foreign direct investment (FDI) increases in emerging economies. The reason behind this is that foreign investors could help intensify the physical capacity in the health care sector, by increasing financial support for diagnostic facilities, number of hospital beds, and increasing the supply of specialty. Again, FDI could provide an upgraded healthcare resources and technology as their corporate social responsibility. These occur in numerous developing countries where multinational corporations built fully equipped health facilities. The interaction effect of labor force and FDI (LAB*FDI) contributes to an increase in public health expenditure. The interaction effect of FDI and labor force in emerging economies will increase individual incomes including the government. This could expand the government revenue base and therefore resources allocated to the health sector is likely to increase as well [42].

From this study, it is evident that healthcare costs is affected by macroeconomic indicators such as economic growth, tax revenue, inflation and labor force have a significant impact on in emerging countries. For instance, an increase in GDP per capita shows an improvement in economic growth of emerging nations and therefore governments can accrue enough tax revenue to boost their revenue base. This gives countries the opportunity to increase their budgets for their health systems by boosting healthcare costs. Similarly, with stable inflation, prices of goods and services including those related to healthcare is stable within a country and hence, governments can forecast accurately future healthcare costs. In totality, the macroeconomic indicators do not only influence the policies of governments’ economic growth but also the health sector including the healthcare cost of emerging economies. For an in-depth understanding of the direction of the association between public health costs and its determinants, the study used the panel causality technique established by [43]. This technique is suitable for heterogeneous and unbalanced panels.

**Figure 3:**
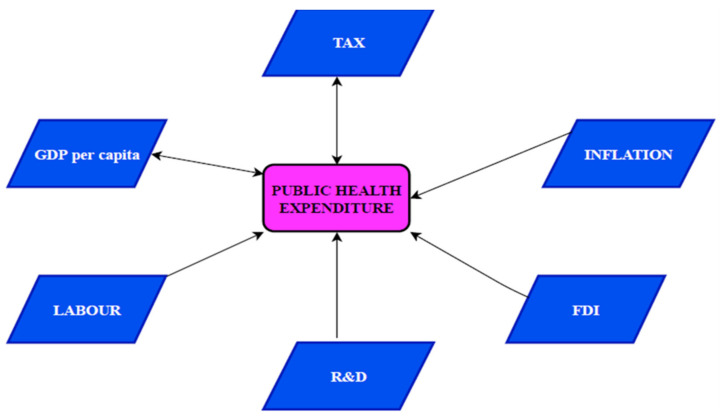
Results of Dumitrescu–Hurlin panel causality test.

In Figure 3, the study describes the findings of the Dumitrescu-Hurlin pairwise panel causality test. The test evidences a bidirectional causal relationship ranging from public health cost to tax revenue and public health cost to GDP per capita, respectively. However, unidirectional causal connections from public health cost to labor force participation, R&D to public health cost, FDI to public health cost and inflation to public health cost, respectively were confirmed. 

## 5. Conclusions

Most countries acknowledge health care as an essential human right of the individual. Governments are required to intercede proactively to enhance access to health care of their citizen’s whiles protecting consumers against direct health costs. The study investigated the effect of macro-economic indicators on public health expenses within emerging economies. Using the generalize method of moments (GMM) and the panel ordinary least squares (OLS) regression for a robust check, data for the study was derived from the World Bank’s World Development Indicators for twenty-one (21) emerging countries, from 2000 to 2018. The empirical results show that increased in public health care cost is associated with a rise in tax revenue and labor force positively increases. However, the association between public healthcare cost and Inflation revealed a declining relationship. The study confirmed a u-shaped relationship between public health cost and economic growth. Public health spending increases as foreign direct investment (FDI), the interactive term between research and development and cardiovascular disease and cancer (R&D×Mortality) rise.

The findings of this study show that healthcare cost is directly affected by macroeconomic indicators because an increase in income levels of a country allows governments to increase their revenue through tax. Public healthcare costs is influenced by Inflation because it affects prices of goods and services including costs of health-related materials. Although labor force participation could increase government tax base, some jobs also expose the workers and the population in general to health risk ultimately resulting in increased healthcare cost. This means public healthcare cost is directly or indirectly associated with macroeconomic indicators in emerging economies.

Furthermore, towards improving public health spending, increasing the fiscal capacity of emerging countries by widening the tax base through the extension of the collection of the domestic tax revenue is essential. Increasing taxes on unhealthy products to serve as a double-edged sword to deter consumers from consuming unhealthy diets can maintain the populations’ health while governments at the same time earn funds to save lives and enhance the general well-being of the public. Another suitable approach to maintaining the populations’ health in emerging countries should include policies to reduce consumption of unhealthy diets, as well as a deliberate nationwide culture of engaging in routine healthy physical activities that will prevent non-communicable diseases.

Governments should also implement strict policies to ensure the wise utilization of revenue collected from taxes. This will contribute to resource availability for health care and improved access to quality health services.

Inflation may reduce public healthcare costs among emerging economies. Given this, governments can engage in the implementation of monetary policies to control inflation by reducing imported goods and enhancing the patronage of locally manufactured products.

This study did not employ other variables such as urbanization rate and foreign aid in the analysis due to the unavailability of data. This, however, does not undermine the findings of the study. In future studies urbanization rates, foreign aid and other variables if available will be included for analysis.

## Figures and Tables

**Table 1:** Data sources and definition.

Abbreviation	Variable Name	Unit	Source
HCE	Public health care expenditure	current international $	WDI
TAX	Tax revenue	current international $	WDI
GDPPC	GDP per capita	constant 2010 US$	WDI
INF	Inflation consumer prices	annual%	WDI
LAB	Labor force participation	Population	WDI
R&D	Research and development expenditure	constant 2010 US$	WDI
Mortality	Death from cardiovascular diseases, cancer, diabetes	Population between exact ages 30 and 70	World Health Organization
FDI	Foreign direct investment, net inflows	constant 2010 US$	WDI

**Table 2:** Descriptive statistics.

Statistics	lnHCE	lnMortality	lnFDI	lnGDPPC	lnLFP	lnTAX	lnR&D
Mean	23.482	6.054	22.536	24.048	17.122	24.335	21.177
Median	23.412	1.000	22.703	23.959	16.916	24.348	21.125
Maximum	25.724	24.800	26.396	26.150	20.487	26.512	23.849
Minimum	21.263	0.055	0.000	21.708	14.381	17.062	18.536
Std. Dev.	0.867	9.417	1.871	0.936	1.407	1.413	1.317
Skewness	0.333	1.227	−4.605	0.256	0.634	−1.818	0.060
Kurtosis	3.346	2.536	55.519	2.876	3.100	8.870	1.886
Jarque–Bera	9.297	102.886	46,910.37	4.596	26.692	786.758	20.716
Probability	0.010	0.000	0.000	0.100	0.000	0.000	0.000
Mortality	3.692	155.544	8.304	3.890	8.217	5.807	6.217

**Table 3:** Panel results for the generalized method of moments model.

	Model 1		Model 2		Model 3
Variable	Coefficient	Variable	Coefficient	Variable	Coefficient
Dep_t-1_	0.102 **	Dep_t-1_	0.517 ***	Dep_t-1_	0.202 *
	(0.050)		(0.182)		(0.075)
lnGDPPc	1.096 ***	lnGDPPc	−0.0262	lnINF	−0.08 ***
	(0.019)		(0.32)		(0.02)
lnMortality	0.0943 ***	lnGDPPc2	0.0234 ***	lnTAX	0.108 ***
	(0.016)		(0.00661)		(0.014)
lnFDI	0.003 *	lnMortality	0.0271 ***	lnLAB*FDI	0.025 ***
	(0.002)		(0.006)		(0.002)
lnR&D	0.007	lnINF	−0.03	lnR&D*Mortality	0.246 ***
	(0.010)		(0.05)		(0.0556)
		lnLAB	0.108 ***	lnFDI*RD	0.29 ***
			(0.027)		(0.07)
		lnTAX	0.00211		
			(0.006)		
Constant	−3.418 ***	Constant	9.090 **	Constant	2.824 ***
	(0.3)		(3.819)		(0.6)
F-stats	2.20 *	F-stats	8.12 **	F-stats	9.01 **
AR-1(Z)	−1.94 **	AR-1(Z)	−1.90 **	AR-1(Z)	−2.44 **
AR-2 (Z)	−0.68	AR-2 (Z)	−1.03	AR-2 (Z)	−052
Hansen-J test	0.202	Hansen-J test	0.206	Hansen-J test	0.213

Standard deviation is in parenthesis. ***, **, * represents 1%, 5% and 10% significance level, respectively.

**Table 4:** Panel results for ordinary least squares regression model.

	Model 1		Model 2		Model 3
Variable	Coefficient	Variable	Coefficient	Variable	Coefficient
lnGDPPC	0.9387 ***	lnGDPPC	−0.0049 ***	lnINF	−0.21 ***
	(0.0231)		(0.0007)		(0.05)
lnMortality	−0.0090 ***	lnGDPPC2	1.0806 ***	lnTAX	0.769 ***
	(0.0020)		(0.0236)		(0.0166)
lnFDI	0.0083	lnMortality	1.0310 ***	lnLAB×FDI	0.0010 ***
	(0.0106)		(0.1083)		(0.0003)
lnR&D	0.036 *	lnINF	−0.07 **	lnR&D×Mortality	0.0558 ***
	(0.0219)		(0.04)		(0.0146)
		lnTAX	0.57 ***	lnFDI×R&D	0.0099 ***
			(0.17)		(0.0008)
		lnLAB	0.42 ***		
			(0.16)		
R-squared	0.8412	R^2^	0.8693	R^2^	0.7600
Adj. R^2^	0.8399	Adj. R^2^	0.8676	Ad. R^2^	0.8700

Standard deviation is in parenthesis. ***, **, * represents 1%, 5% and 10% significance level, respectively.
